# Thermal niche evolution and geographical range expansion in a species complex of western Mediterranean diving beetles

**DOI:** 10.1186/s12862-014-0187-y

**Published:** 2014-09-04

**Authors:** Amparo Hidalgo-Galiana, David Sánchez-Fernández, David T Bilton, Alexandra Cieslak, Ignacio Ribera

**Affiliations:** Institute of Evolutionary Biology (CSIC-Universitat Pompeu Fabra), Passeig Maritim de la Barceloneta 37-49, 08003 Barcelona, Spain; Marine Biology and Ecology Research Centre, Plymouth University, Drake Circus, PL4 8AA Plymouth, UK

**Keywords:** Thermal niche evolution, Cold tolerance, Demographic expansion, Dytiscidae, Western Mediterranean

## Abstract

**Background:**

Species thermal requirements are one of the principal determinants of their ecology and biogeography, although our understanding of the interplay between these factors is limited by the paucity of integrative empirical studies. Here we use empirically collected thermal tolerance data in combination with molecular phylogenetics/phylogeography and ecological niche modelling to study the evolution of a clade of three western Mediterranean diving beetles, the *Agabus brunneus* complex.

**Results:**

The preferred mitochondrial DNA topology recovered *A. ramblae* (North Africa, east Iberia and Balearic islands) as paraphyletic, with *A. brunneus* (widespread in the southwestern Mediterranean) and *A. rufulus* (Corsica and Sardinia) nested within it, with an estimated origin between 0.60-0.25 Ma. All three species were, however, recovered as monophyletic using nuclear DNA markers. A Bayesian skyline plot suggested demographic expansion in the clade at the onset of the last glacial cycle. The species thermal tolerances differ significantly, with *A. brunneus* able to tolerate lower temperatures than the other taxa. The climatic niche of the three species also differs, with *A. ramblae* occupying more arid and seasonal areas, with a higher minimum temperature in the coldest month. The estimated potential distribution for both *A. brunneus* and *A. ramblae* was most restricted in the last interglacial, becoming increasingly wider through the last glacial and the Holocene.

**Conclusions:**

The *A. brunneus* complex diversified in the late Pleistocene, most likely in south Iberia after colonization from Morocco. Insular forms did not differentiate substantially in morphology or ecology, but *A. brunneus* evolved a wider tolerance to cold, which appeared to have facilitated its geographic expansion. Both *A. brunneus* and *A. ramblae* expanded their ranges during the last glacial, although they have not occupied areas beyond their LGM potential distribution except for isolated populations of *A. brunneus* in France and England*.* On the islands and possibly Tunisia secondary contact between *A. brunneus* and *A. ramblae* or *A. rufulus* has resulted in introgression. Our work highlights the complex dynamics of speciation and range expansions within southern areas during the last glacial cycle, and points to the often neglected role of North Africa as a source of European biodiversity.

**Electronic supplementary material:**

The online version of this article (doi:10.1186/s12862-014-0187-y) contains supplementary material, which is available to authorized users.

## Background

Information on the thermal biology of a species is fundamental to understand its ecology, biogeography and evolution, as species are only capable of tolerating a limited range of climatic conditions. Ambient temperature affects all biological processes [1,2], especially in ectotherms [3], and is usually assumed to be one of the main determinants of their spatial distribution [4]. However, in most biogeographical studies the thermal tolerance of species is extrapolated exclusively from their current distributions [5], and even when palaeoclimatic or genetic data are considered (as in e.g. [6–8]), it is rare for these to be combined with experimental data on the actual physiological tolerance of the study organisms [9,10]. Despite this, the need for integrative approaches is increasingly being recognised [11–13], particularly given the limitations of current distributional data for inferring historical or ecological processes [14,15].

Here we attempt such an integrative approach in a clade of diving beetles that has diversified and expanded its range in the western Mediterranean region during the late Pleistocene, the *Agabus brunneus* complex [16]. Previous work has revealed that thermal tolerance is a good predictor of geographical range extent in these beetles, in which more widespread species have wider thermal windows than their narrow-range relatives [17]. Two species of the complex have partly overlapping distributions in southwest Europe and North Africa, whilst the third is confined to the islands of Corsica and Sardinia [16].

Traditionally, the study of the recent evolutionary history of the European fauna and flora has largely considered the direct effect of the Pleistocene glaciations, particularly the recolonization of previously glaciated areas from unglaciated refugia and the genetic changes resulting from such range movements [18–20]. In most cases, unglaciated areas are simply seen as refugia for northern species, little attention being paid to evolutionary and biogeographical processes in them, other than those which affected these species [21,22]. In contrast to this view, the current diversity of the Mediterranean area is increasingly seen to result from processes which are not directly related to the range movements of northern species during glacial-interglacial cycles [23–26], but our understanding of its origin remains fragmentary, particularly for highly speciose groups such as most insects.

In this study we integrate current and palaeoclimatic information with a molecular phylogeography, morphological analysis and experimentally derived thermal tolerance data to understand the role of thermal niche differences in shaping geographical expansion and speciation processes within the *A. brunneus* complex. Our specific goals are to: 1) test for climatic niche divergence among these species, and associate these differences with their current distribution; 2) test for differences in the estimated ecological niche of each species, and reconstruct the changes in their potential distributions through the last glacial cycle; and 3) evaluate species limits and reconstruct the speciation processes, demographic evolution and range expansion within the *A. brunneus* complex.

We use mitochondrial and nuclear sequence data from populations throughout the extant geographical ranges of all three species of the complex to reconstruct their demographic history and geographic expansion, and explore these within the context of changes in estimated potential distributions through the last glacial cycle. Using the current distribution of the species, we test for differences in climatic niche, and contrast these with experimental data obtained from range edge populations. By integrating these diverse data we are able to reconstruct the evolutionary history of the *A. brunneus* complex in the southwestern Mediterranean region, and illustrate how late Pleistocene climate changes may have shaped its current diversity by promoting ecological differentiation within a southern refuge.

## Methods

### Taxonomic background on the *Agabus brunneus* complex

**Figure 1:**
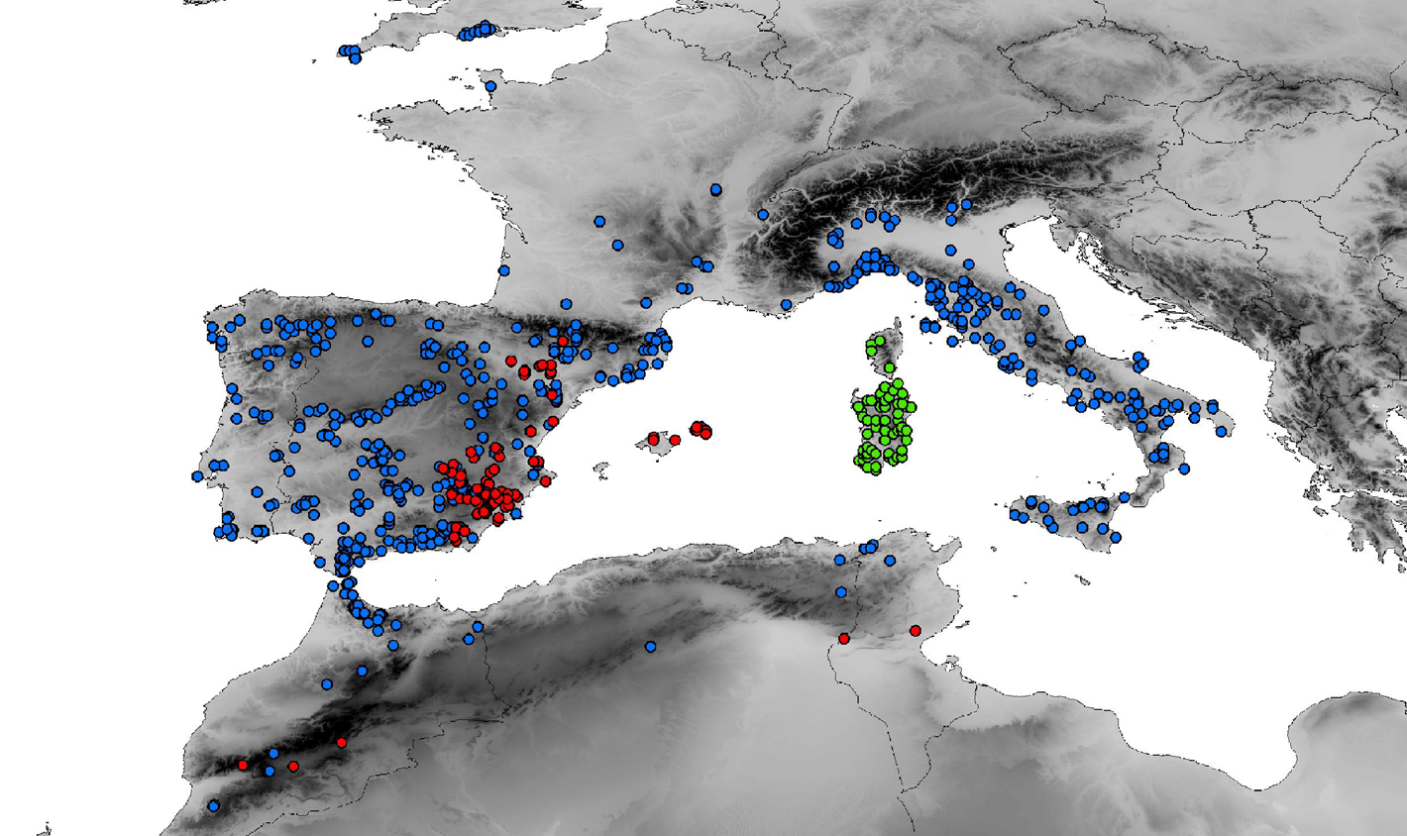
**Known distribution of the species of the** ***Agabus brunneus*** **complex, including all localities used for DNA sequencing and ecological niche modelling.** Blue circles, *A. brunneus*; red circles, *A. ramblae*; green circles *A. rufulus.*

The *Agabus brunneus* complex (Coleoptera, Dytiscidae) includes three recognised species of diving beetles with a distribution centred in the western Mediterranean area [16]: *Agabus brunneus* (Fabricius, 1798), *A. rufulus* Fairmaire, 1860 and *A. ramblae* Millán & Ribera [16]. Together with the more distantly related *A. didymus* (Olivier, 1795), which is more widely distributed in the western Palaearctic, they form the *Agabus brunneus* group [27,28]. *Agabus brunneus* has a wide distribution through North Africa and western Europe, including the Iberian and Italian peninsulas, some Mediterranean islands (Elba, Sicily) and France, with isolated populations in southern England ([16]; Figure 1). Some old, isolated records in the eastern Mediterranean (Greece, Syria) with all probability refer to other species (e.g. *A. dilatatus* (Brullé, 1832), unpublished observations). *Agabus ramblae* was recognised based on external morphology and male genitalia as a distinct species previously confounded with *A. brunneus*, and has a disjunct distribution in the South and East of the Iberian Peninsula, the Balearic Islands, Central Morocco and Tunisia [29]. It is usually found in mineralized, temporary running waters [16], and despite its more recent description, exhaustive re-examination of material of *A. brunneus sensu lato* suggests that its apparently restricted range is genuine. The third species, *A. rufulus*, was traditionally considered a colour variety of *A. brunneus* and recorded from various localities in Italy (including the Tyrrhenian islands), Spain and North Africa [30,31]. The revision of Millán & Ribera [16] revealed that it is, instead, a Corsico-Sardinian endemic. *Agabus ramblae* and *A. rufulus* have fully allopatric distributions, but the range of *A. brunneus* completely overlaps with that of *A. ramblae*, and it has also been recorded in Corsica and Sardinia. *Agabus brunneus* and *A. ramblae* are very rarely syntopic, with only 10 reported co-occurrences in the same locality (9 in the province of Albacete and 1 in the nearby area of Jaén, A. Millán, personal communication, 2014). Prior to this study very limited mitochondrial data were available for the three species [32,33], and details of their phylogenetic/phylogeographic relationships, or age of divergence, were lacking.

### Morphological identification of species

The main diagnostic difference among species of the *A. brunneus* complex is the shape of the median lobe of the male aedeagus, in particular its relative size, the degree of asymmetry in ventral view and the shape of the apex in lateral view [16], and it is this character suite which was primarily used to assign material to species here. Female specimens were assigned to species by association with males and according to body dimensions and colouration. For 121 males of the three species we measured the maximum length of the median lobe of the aedeagus (AL) and the asymmetry of the aedeagus in ventral view (AD), as these are the main characters separating *A. brunneus* from *A. ramblae*, the two coexisting species [16]. We measured asymmetry (AD) as the difference between the width of the right (RD) and left (LD) sides in the point of its maximum width. We also measured the total body length from the anterior margin of the pronotum to the apex of the elytra (BL), as well as the maximum body width at the widest point (BW) (Additional file 1: Figure S1; Additional file 2: Table S1). Measured males included all specimens used for DNA sequencing (see below) and additional material from a number of sources, including areas for which no fresh material could be obtained (such as Mallorca). Species of the genus *Agabus* have a very uniform shape, and the length of a specimen is a good surrogate of total size [34]. For both *A. brunneus* and *A. ramblae* there is no difference between the body length of males and females [16]. We tested for differences in the length and shape of the aedeagus across the three species using MANOVA, and checked for possible intraspecific geographical variation within *A. ramblae* from Morocco, the Iberian Peninsula and the Balearic Islands, and *A. rufulus* from Corsica and Sardinia. All analyses were performed in IBM SPSS Statistics v. 20 (IBM, Armonk, NY, US).

### Thermal tolerance

We determined the thermal tolerance of two populations of different species (Additional file 3: Table S5), one of *A. brunneus* from NE Spain (Girona, Ser river 42°08'48''N 2°34'48''E) and one *A. ramblae* from central Morocco (Tinghir, Toudgha river 31°33'25''N 5°34'49''W). The experimental procedure followed that used by Calosi et al. [17], from which we extracted data for three additional populations of three different species (*A. brunneus*, *A. ramblae* and *A. rufulus*). Individuals were acclimated for 7 days at 14.5°C in controlled conditions of pH, water composition, light regime and food (red chironomid larvae). Specimens were then separated into sub-groups and thermally ramped (±1°C min^−1^) in a computer-controlled water bath (Grant Instruments Ltd, Herts, UK) to obtain measures of their Upper Thermal Limit (UTL) and Lower Thermal Limit (LTL). Temperature was directly measured in one of the wells where individuals were placed for the experiment with a calibrated digital thermometer (Omega HH11; Omega Engineering Inc., Stamford, CT, USA) (see [17] for details). Data were analysed with an ANOVA with species and population as factors and DHS or Tukey post-hoc tests using IBM SPSS Statistics v. 20.

### Taxon sampling, DNA extraction and sequencing

Specimens were collected in the field and directly preserved and stored in absolute ethanol. We included molecular data from 68 populations of *A. brunneus*, 22 *A. ramblae* and 6 *A. rufulus*, with up to five individuals per location when available, giving a total of 203 sequenced individuals covering the entire geographical ranges of the three species (Figure 1; Additional file 4: Table S2). As outgroups we used *A. didymus* (the sister of the *A. brunneus* complex [16,27]) together with other published sequences representing a wide range of genera/species of Agabini (Additional file 4: Table S2). Trees were rooted in the genus *Platynectes*, which is within the Agabini but clearly outside the *Agabus* group of genera [32,35,36].

For DNA isolation we employed commercial DNA tissue kits (Additional file 4: Table S2) following the manufacturer instructions. Voucher specimens and DNA aliquots are deposited in the Natural History Museum (NHM, London), Museo Nacional de Ciencias Naturales (MNCN, Madrid) and Institute of Evolutionary Biology (IBE, Barcelona) (Additional file 4: Table S2).

To define the closest outgroups and the general time frame of diversification we used a combination of mitochondrial (a fragment of 827 nucleotides at the 3’ end of *cox1*, a continuous fragment between 798–803 (Agabini) or 802 nucleotides (*A. brunneus* complex) including the 3’ end of *rrnL* + full *trnL* + 5’ end of *nad1*) and two fragments of the nuclear genes *SSU* and *H3* of 608 and 327 nucleotides respectively (see Additional file 5: Table S3 for the primers used and the general PCR conditions). For some specimens in which the *cox1* fragment could not be amplified in a single PCR we used internal primers to obtain two non-overlapping fragments of ca. 400 bp each.

For the detailed phylogeographic and coalescent analyses we sequenced two gene fragments, one mitochondrial (3’-*cox1*) and one nuclear (*H3*). Sequence errors/ambiguities were edited using Geneious Pro 5.3.6 (http://www.geneious.com). New sequences have been deposited in GeneBank with accession numbers LM654767-LM655064 and LM655068-LM655168 (Additional file 4: Table S2).

### Phylogenetic analyses

Length-variable sequences were aligned with the on-line version of MAFFT v.7 [37] using the Q-INS-i algorithm, which considers the secondary structure of RNA, and default values for other parameters. The final aligned matrix for the analyses of Agabini was 2579 nucleotide long.

#### General phylogenetic relationships

To determine the relationships among the main lineages within the *A. brunneus* complex and its phylogenetic relationships within Agabini we used the combined mitochondrial and nuclear sequence from a selection of specimens of the three species. Specimens were selected to cover the geographic range of all species, with a particular focus on potential contact areas (identified through preliminary analyses). Analyses used Bayesian probabilities as implemented in BEAST v1.7 [38] with a partition by genes (except for the *trnL*, pooled with the *rrnL* fragment) and a GTR + I + G evolutionary model for each partition. BEAST was run for 100 million generations, with 10% considered as the burn-in fraction after checking convergence of all parameters with the effective sample size (ESS) as measured in TRACER v1.5 [38].

To establish a temporal framework for the origin and evolution of the *A. brunneus* complex we used the mitochondrial genes only, for which there are recent calibrations for different families of Coleoptera with very homogeneous estimations for the rate of a combination of protein coding and ribosomal mitochondrial regions, calibrated with fossils and different biogeographic events [39–41]. As a prior we used a normal distribution with an average combined rate of 0.01 substitutions/site/million years (MY) and a standard deviation of 0.001, with other settings identical to the above analysis. To ensure that we obtained the same topology as in the analysis employing the full sequence, we constrained the monophyly of ingroup and outgroup, the genera and the *A. brunneus* complex. We used TRACER to calculate the mean and 95% highest posterior density interval for divergence times. We tested different alternative topologies for the relationships amongst species of the *A. brunneus* complex via the use of Bayes factors as estimated with the stepping-stone (SS) and the path-sampling (PS) algorithms in BEAST [42], and with the harmonic mean estimator (HME) in TRACER 1.5 [43] for comparison, in this case requiring an improvement in marginal likelihood of 10 units per additional parameter before accepting a more complex model [44,45]. We tested three different topologies: 1) unconstrained (C0); 2) respective monophyly of *A. brunneus* and *A. ramblae* (C1); and 3) monophyly of *A. rufulus* (C2). We also analysed the *H3* sequences separately in BEAST and RAxML, using a range of outgroups (Additional file 4: Table S2), a single partition with a GRT + G evolutionary model and the previously estimated age of the genus *Agabus* as prior to calibrate the tree in BEAST.

To test for alternative demographic models and to establish haplotype distribution and relationships we used the mitochondrial gene *cox1* of all sequenced specimens of the *A. brunneus* complex, with the same settings as described for the analyses above, but with a mean rate of 0.02 (following [39,40]), with a standard deviation of 0.001. Demographic models were tested first with the haplotypes of the *A. brunneus* complex only (i.e. excluding *A. didymus*), without topological constraints, and then with the haplotypes of *A. brunneus* only (i.e. also excluding *A. ramblae* and A*. rufulus*) (Additional file 4: Table S2).

Four models were tested: 1) constant population size; 2) exponential growth; 3) expansion; and 4) logistic growth. Models were compared through Bayes factors as above, i.e. using the HME, PS and SS. We also constructed a Bayesian skyline plot (BSP, [46]) with the combined results of two independent analyses of the *A. brunneus* complex in TRACER v1.5. The BSP constructs a model of demographic history based on how the number of coalescent events over a given interval differs from that expected under a neutral model for a panmictic population, then summarizes all possible genealogies and provides confidence intervals for all parameters in the model. It estimates changes in effective population size to analyse the population expansion of a species.

#### Population genetic analysis

We estimated some measures of haplotype diversity and analysed raggedness indices for demographic expansion with Arlequin 3.5 [47] using the *cox1* gene. We tested the validity of the estimated stepwise expansion model using a parametric bootstrap approach.

### Ecological niche modelling

We used ecological niche modelling (ENM) based on large-scale climatic data sets and known occurrence points to characterise the environmental niche of all three species, and to test for niche divergence amongst them. Climatic data were obtained at a spatial resolution of approximately 0.08° from WORLDCLIM version 1.3 (19 bioclimatic variables from http://www.worldclim.org; [48]; Additional file 6: Table S4). As records of species occurrences, we employed all known localities for the *A. brunneus* species complex identified according to the morphology of the males (Additional file 7: Table S6) at the same resolution than the bioclimatic variables. Most of the localities used in the ENM were also represented in the molecular analyses.

Bioclimatic values were first subjected to a principal component analysis (PCA) to obtain uncorrelated environmental factors (Varimax rotation). We used the values of the first two PCA factors to represent the climatic space of the whole study area. We plotted the occurrences of the three species in this same space, to visualize the section of the climatic space occupied by each species.

As the results of the thermal tolerance experiments clearly pointed to the importance of lower thermal limits, we used a Kruskal-Wallis ANOVA to compare the minimum temperatures of the coldest month between the localities of the three species. Multiple comparison tests were used to detect significant differences between means. The PCA and the Kruskal-Wallis ANOVA were conducted in Statistica version 8.0 (www.statsoft.com, 2007).

To compare the climatic niche of species we generated ecological niche models using MaxEnt [49], with Schoener’s D [50] as a measure of niche similarity between each pair of species as calculated by ENMTOOLS [51]. These values were calculated by comparing the climatic suitability of each grid cell in the study area obtained with MaxEnt. As niche differences may be simply a result of the spatial autocorrelation of the explanatory environmental variables [52], we conducted a background similarity test, also implemented in ENMTOOLS. This test uses randomization to determine whether two species are more or less similar than expected based on the differences in the environmental background in which they occur. A null distribution of 100 niche similarity values was generated by comparing the model suitability values of one species to those generated from random cells drawn from the distribution of the other species. The observed D value of niche similarity between the two species was then compared with the null distribution generated for each of them. The background area of each species should be adjusted to the habitat available, and should be biologically realistic [51]. For the insular *A. rufulus*, the background area was geographically restricted to Corsica and Sardinia, and for *A. ramblae* and *A. brunneus* we defined the background area as the Freshwater Ecoregions in which each species occurs following the classification in Abell et al. [53] (Additional file 8: Figure S2). This method has been used in a number of studies (e.g. [54,55]), including aquatic Coleoptera [56,57].

To provide a climatic context for the interpretation of the demographic models and the changes in distribution of species, we estimated the potential distribution of *A. brunneus* and *A. ramblae* for current climatic conditions, the reconstructed conditions during the last glacial maximum (LGM, 21,000 years before present, YBP) and the last interglacial (LIG, ca. 120,000-140,000 YBP). To estimate potential (not realized) distributions we used a multidimensional-envelope, as it provides a better estimate from observed occurrences (see [58] for details, Additional file 9: Text S1). For both past scenarios we used the same 19 bioclimatic variables at the same resolution as for current climate (see above). For the LGM we used a simulation of the general circulation model (GCM) from the Community Climate System Model (CCSM, http://www.ccsm.ucar.edu/, [59]). The original GCM data were downloaded from the PMIP2 website (http://www.pmip2.cnrs-gif.fr/). For the LIG we used data provided by Otto-Bliesner et al. [60], available at www.worldclim.org.

## Results

### Morphology

The three species differed in body size, as measured with BL and BW (MANOVA, Roy’s greatest root F = 292.134, *P* < 0.001), with pairwise differences being significant for all comparisons (Table 1). *Agabus brunneus* was the largest of the three species, and *A. ramblae* the smallest. The measures of size and asymmetry of the aedeagus (AL and AD respectively) were also significantly different (MANOVA, Roy’s greatest root F = 513.120, *P* < 0.001, Table 1). *Agabus ramblae* and *A. brunneus* were fully separated by AD, with no intermediate specimens, whilst the specimens identified as *A. rufulus* had an intermediate, overlapping shape (Additional file 10: Figure S3). For both *A. ramblae* (comparing Morocco, Iberian peninsula and the Balearic Islands) and *A. rufulus* (Corsica and Sardinia) there were no significant differences between major geographical areas.

**Table 1:** **Comparison of the measurements of body and male genitalia (aedeagus) among the three species of the** ***A. brunneus*** **complex**

**A) General MANOVA**					
**Variables**	**Effect**	**Value**	***F***	**Sig.**	**Power**
BL, BW (DVs); species (IVs)	Pillai trace	0.85	41.95	<0.001	1
	Wilks’ lambda	0.16	84.12	<0.001	1
	Hotelling trace	5.14	143.82	<0.001	1
	Roy’s greatest root	5.13	292.13	<0.001	1
AL, AD (DVs); species (IVs)	Pillai trace	0.96	50.62	<0.001	1
	Wilks’ lambda	0.09	125.66	<0.001	1
	Hotelling trace	9.48	253.53	<0.001	1
	Roy’s greatest root	9.42	513.12	<0.001	1
**B) Pairwise ** ***t*** **comparisons**					
**t-test comparison**	**BL**	**BW**	**AL**	**AD**	
*A. brunneus* vs *A. ramblae*	<0.001	<0.001	<0.001	<0.001	
*A. brunneus* vs *A. rufulus*	<0.001	<0.001	<0.001	<0.001	
*A. ramblae* vs *A. rufulus*	<0.001	<0.001	<0.001	0.1	
C) Mean values					
	BL	BW	AL	AD	
*A. brunneus*	7.75	4.86	1.44	0.07	
*A. ramblae*	6.67	4.30	1.15	0.01	
*A. rufulus*	7.32	4.57	1.33	0.02	

BL, body length; BW, body width; AL, length of the aedeagus; AD, asymmetry of the aedeagus (see Additional file 1: Figure S1). DVs, dependent variables; IVs, independent variables.

### Thermal tolerance

**Figure 2:**
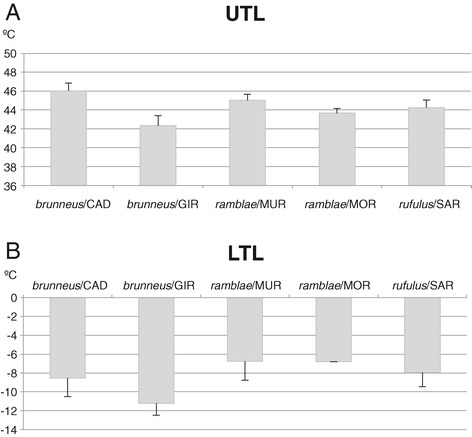
**Results of thermal tolerance experiments on the five populations tested. A)** upper thermal limit (UTL), **B)** lower thermal limit (LTL). CAD, Cádiz; GIR, Girona; MUR, Murcia; MOR, Morocco; SAR, Sardinia.

Overall differences in thermal tolerance were highly significant between populations for both LTL (F = 9.87, d.f. = 4, *P* < 0.001) and UTL (F = 27.2, d.f. = 4, *P* < 0.001; Figure 2, Table 2, Additional file 3: Table S5). For UTL the highest differences were between the two populations of *A. brunneus* (north and south Iberian peninsula, post-hoc Tukey *P* < 0.001; higher limit in the southern population), which were also different for LTL (post-hoc Tukey *P* < 0.01; lower limit in the northern population). Differences between the two populations of *A. ramblae* (Morocco and SE Spain) were significant for UTL (post-hoc Tukey *P* < 0.05; higher limit in the northern population) but not for LTL (Figure 2; Table 2).

**Table 2:** **Differences in thermal tolerance among the species and populations of the** ***A. brunneus*** **complex**

**A) General ANOVA**								
	**Comparison**	**Sum of squares**	**d.f.**	**Quadratic mean**	**F**	**Sig.**	**Power**	
UTL	species	2.1	2	1.0	0.46	0.6	0.12	
	population	72.1	4	18.0	27.20	<0.001	1.00	
LTL	species	77.8	2	38.9	10.90	<0.001	0.99	
	population	113.8	4	28.4	9.87	<0.001	1.00	
**B) post-hoc Tukey**								
							**c.i. (95 %)**	
	**Comparison**	**sp (I)**	**sp (J)**	**Mean difference (I-J)**	**Std.e.**	**Sig.**	**Lower limit**	**Upper limit**
UTL	species	1	2	−0.42	0.50	0.7	−1.63	0.79
			3	0.04	0.57	1.0	−1.33	1.41
		2	3	0.46	0.58	0.7	−0.94	1.86
	population	1	2	3.69	0.37	<0.001	2.62	4.75
		3	4	1.37	0.47	<0.05	0.04	2.69
LTL	species	1	2	−2.96	0.65	< 0.001	−4.53	−1.40
			3	−1.80	0.69	<0.05	−3.47	−0.13
		2	3	1.16	0.73	0.26	−0.61	2.94
	population	1	2	2.70	0.76	<0.01	0.52	4.87
		3	4	0.08	10.96	1.0	−3.11	3.14

In “comparison”, “species” refer to the comparison of the three species (pooling the two populations of *A. ramblae* and *A. brunneus* respectively; 1, *A. brunneus*; 2, *A. ramblae*; 3, *A. rufulus*); “population” refers to the comparison of the five populations (1, *A. brunneus* from south Spain, Cádiz; 2, *A. brunneus* from northeast Spain, Girona; 3, *A. ramblae* from southeast Spain, Murcia; 4, *A. ramblae* from central Morocco, Tinghir). UTL, upper thermal limit; LTL, lower thermal limit; d.f., degrees of freedom; Sig., significance; Std.e, standard error; c.i. confidence interval.

Between species there were significant differences in LTL (*F* = 10.9, d.f. = 2, *P* < 0.001; Figure 2; Table 2; lower limit in *A. brunneus*, higher in *A. ramblae*), but not UTL. Post-hoc analyses for LTL were highly significant in the case of *A. brunneus* vs. *A. ramblae* (*P* < 0.001) and *A. brunneus* vs. *A. rufulus* (*P* < 0.05), but not for *A. ramblae* vs *A. rufulus* (Table 2).

### Phylogeny and phylogeography

#### Phylogenetic placement of the A. brunneus complex

There were no length differences in the mitochondrial and nuclear protein coding genes (*nad1*, *cox1* and *H3*), and amongst the ribosomal genes length differences were restricted to outgroups, with a maximum difference of 2–5 bp. There were few amino-acid changes in the protein coding genes within the *A. brunneus* complex, and only two in the *H3* fragment: one shared by all *A. rufulus* with the exception of one specimen from Sardinia (AH223), and another shared by all *A rufulus* and all *A. brunneus*.

**Figure 3:**
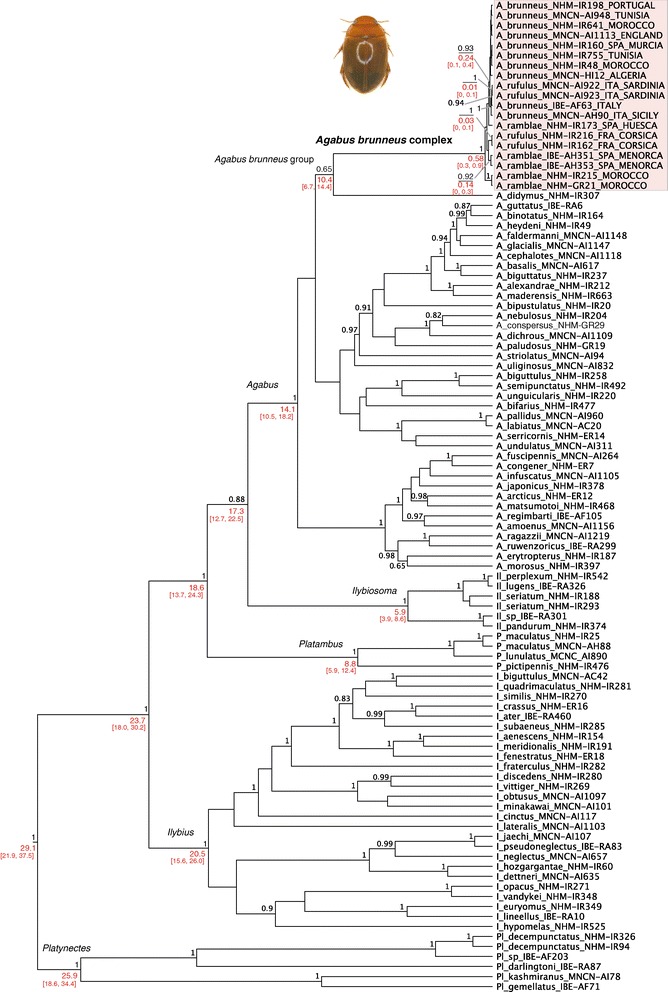
**Phylogenetic relationships of the** ***Agabus brunneus*** **complex, obtained with BEAST and the combined mitochondrial and nuclear sequence.** Numbers in black, node posterior probabilities; numbers in red, estimated age of the nodes (Ma) and 95% confidence intervals, obtained from the analysis of the mitochondrial data only (see Figure 4 and Additional file 11: Figure S4).

In the Bayesian analysis of the combined data (mitochondrial plus nuclear) the genus *Agabus* was recovered as monophyletic with strong support (Figure 3), and the *A. brunneus* group also monophyletic but with lower support (posterior probability, pp = 0.61) and with a long stem branch. The monophyly of the *A. brunneus* complex was strongly supported (pp = 1), with *A. ramblae* basal and paraphyletic and a polyphyletic *A. rufulus*: two Corsican specimens were sister to Menorcan *A. ramblae*, while two Sardinian specimens were nested within *A. brunneus*.

#### Internal phylogeny of the A. brunneus complex, divergence age estimation

**Figure 4:**
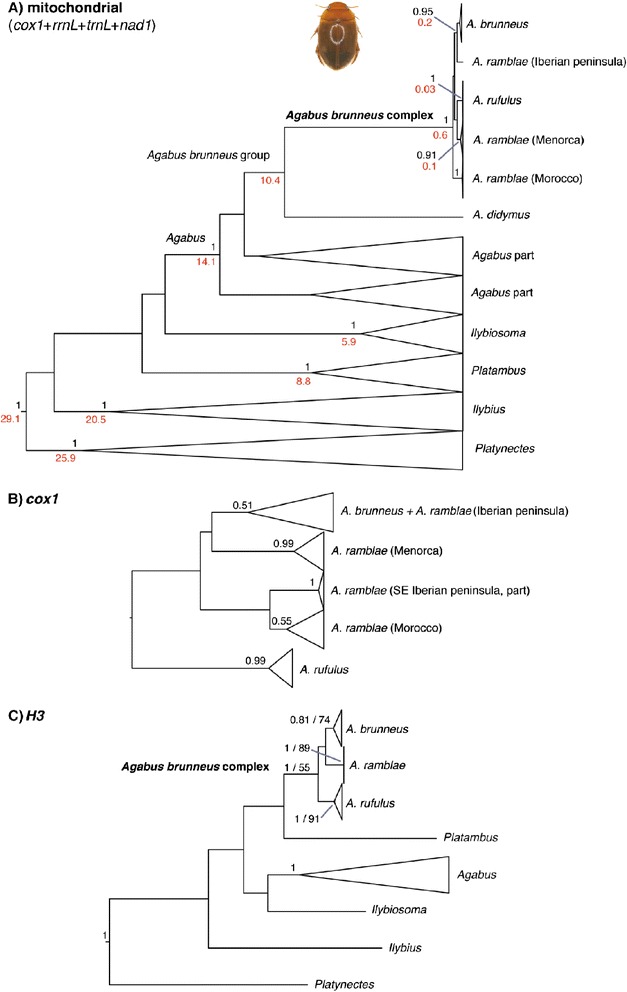
**Summary trees obtained from A) combined mitochondrial data (** ***cox1*** **+** ***rrnl*** **+** ***trnl*** **+** ***nad1*** **), B)** ***cox1*** **and C)** ***H3*** **.** Node supports in **A)** and **B)**, Bayesian posterior probabilities; **C)** Bayesian pp/ML bootstrap values. In red, estimated age of the nodes (see Additional file 11, Additional file 12 and Additional file 13 for the complete topologies of the trees).

The estimation of a time window for the diversification of the *A. brunneus* complex, using the mitochondrial sequence from the above specimens and an a priori rate obtained from related groups, gave an age for the stem group of approximately 10.4 Ma (million years ago). The estimated origin of the sampled diversity within the complex was much more recent (0.6 Ma, Figure 3). Models using the two constraints tested (monophyly of *A. ramblae*, C1, and *A. rufulus*, C2) preformed significantly worse than the unconstrained model (C0), with the monophly of A*. rufulus* being the worst of all for all three Bayes factor estimators (HME, PS and SS; with more than 10 units in the difference in –lnLH for the HME, and more than four units in the case of PS and SS, [61], Table 3). The preferred mitochondrial topology had the Moroccan haplotypes of *A. ramblae* sister to the rest of the complex (range of uncorrected mitochondrial p distances, p = 0.004-0.012), Corsican *A. rufulus* sister to Menorcan *A. ramblae* with a divergence of ca. 0.34 Ma (p = 0.008), and Iberian *A. ramblae* sister to *A. brunneus* + Sardinian *A. rufulus*, with a divergence date of ca. 0.35 Ma (p = 0.002; Figure 4, Additional file 11: Figure S4).

**Table 3:** **Bayes factors for the topological comparisons**

**A)**			
**Constraint**	**HME**	**PS**	**SS**
C0	−23985	−24419	−24421
C1	−24004	−24424	−24425
C2	−24015	−24443	−24444
**B)**			
**Model**	**HME**	**PS**	**SS**
C	−1696	−2052	−2053
Ex	−1729	−2041	−2042
Es	−1734	−2043	−2044
L	−1727	−2036	−2038

A) Topological constraints: C0, no constraint; C1, *A. ramblae* and *A. brunneus* respectively monophyletic; C2, *A. rufulus* monophyletic. B) Demographic models of the *A. brunneus* complex: C, constant population size; Ex, exponential growth; Es, expansion; L, logistic growth.

HME, harmonic mean stimator; PS, path sampling; SS, stepping stone (see text for details).

We also analysed the *cox1* and *H3* sequences independently. The analysis of the *cox1* gene for all sequenced specimens (203, Additional file 4: Table S2), using *A. didymus* as an outgroup, resulted in a topology very similar to that described above, but with some additional Sardinian *A. rufulus* grouped with Corsican specimens and additional Iberian *A. ramblae* grouped with the Moroccan specimens and nested within *A. brunneus* (Figure 4, Additional file 12: Figure S5), in some cases with identical haplotypes. Two specimens of *A. ramblae* from Menorca (AH348, AH352, the later a male with typical *A. ramblae* aedeagus, Additional file 2: Table S1) and one from Morocco (AH311, female) were also nested within *A. brunneus*.

The analysis of the *H3* gene recovered the three species as respectively monophyletic with good support (*A. ramblae* and *A. rufulus* pp = 1, ML bootstrap = 89 and 91 respectively; *A. brunneus* pp = 0.8, MLb = 74; Figure 4, Additional file 13: Figure S6), with only one exception, one female from Albacete identified as *A. ramblae* (AH224) was grouped with *A. brunneus*. Within *A. brunneus* and *A. rufulus* there was some variation (one position), without geographical structure (Figure 4, Additional file 13: Figure S6).

#### Demographic models of the expansion of the A. brunneus complex

We compared four coalescent demographic models in BEAST using only the *cox1* data of the three species within the *A. brunneus* complex. The best model according to Bayes factors using the PS and SS estimators was logistic growth (with a difference of more than four units of –lnLH), followed by exponential growth (Table 3). On the contrary, marginal likelihood (HME) suggested a constant population size model as optimal (with a difference of more than 20 units of –lnLH), followed by logistic growth. The constant population model performed worst for both PS and SS estimators (Table 3).

**Figure 5:**
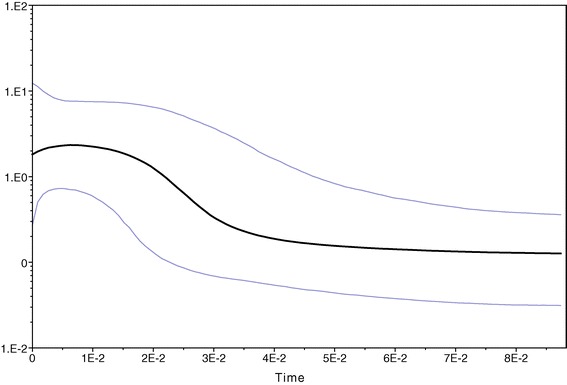
**Bayesian skyline plot of the** ***cox1*** **sequence data of the 203 studied specimens of the** ***Agabus brunneus*** **complex.** Blue lines, 95% highest probability density; horizontal axis, time before present (Ma); vertical axis, effective population size (N_e_T).

The combined two independent runs of the Bayesian skyline plot gave a good convergence when the burn-in fraction was extended to 40 million generations (40%), and suggested exponential population growth at ca. -0.03 Ma, followed by a levelling off at ca. -0.01 Ma with a slight decrease towards the present, i.e. a sigmoidal curve of population growth (Figure 5).

The expansion raggedness indices were lower for populations of *A. brunneus* and *A. rufulus* (from Corsica) than for *A. ramblae*, indicating an expansion in the former two species (Table 4). *Agabus ramblae* also had a higher molecular diversity, as measured with the Theta S and Theta Pi indexes (Table 4).

**Table 4:** **Measures of raggedness and molecular diversity of the three species of the** ***A. brunneus*** **complex**

**Species**	**n. pop.**	**h**	**n.pop. >2 ind**	**n. loci**	**n.loci <5 % mis.**	**pol. sites**	**Ts**	**Tv**	**Θ_S**	**s.d. Θ_S**	**Θ_π**	**s.d. Θ_π**	**RI**
*A. brunneus*	154	28	69	826	717	21	19	2	0.22	0.18	0.22	0.34	0.07
*A. ramblae*	21	10	6	826	766	12	11	1	1.82	1.19	1.81	1.45	0.31
*A. rufulus*	9	2	4	826	762	1	1	0	0.37	0.37	0.35	0.48	0.10

n.pop., number of populations; h, number of haplotypes; n.pop. >2 ind, number of populations with more than two individuals; n.loci <5% mis., number of loci with less than 5% of missing data; pol. sites., number of polymorphic sites; Ts, number of transitions; Tv, number of transversions; Θ_S, estimation of the mutation parameter (Theta) from the observed number of segregating sites (S); s.d. standard deviation; Θ_π, estimation of the mutation parameter (Theta) from the mean number of pairwise differences (Pi); RI, average raggedness index.

### Ecological niche modelling data

The two first axes of the PCA of climatic variables for all localities of the *A. brunneus* complex jointly accounted for 82.4% of the total variance, and were interpreted as representing ‘aridity’ and ‘seasonality’ gradients respectively. The first axis was positively correlated to maximum temperature of the warmest month, and negatively to precipitation of the driest month, whilst the second axis was negatively correlated with temperature seasonality. The environmental space of *A. brunneus* encompassed almost completely that of the other two species: *Agabus ramblae* occupied the more seasonal and arid extreme of the climatic space of *A. brunneus* complex, and *A. rufulus* was climatically close to *A. ramblae*, although in areas with lower aridity and seasonality (Additional file 14: Figure S7).

The values of the minimum temperature of the coldest month of the three species (*A. brunneus*: −8.8°C, *A. ramblae*: −4.8°C, *A. rufulus*: −1.9°C) were significantly different, as measured with a Kruskal-Wallis test (N = 686; H = 73.32, *P* < 0.05). All pairwise comparisons were also significantly different (at *P* < 0.05) except that for *A. brunneus* and *A. ramblae*, which was close to significance (*P* = 0.07).

**Figure 6:**
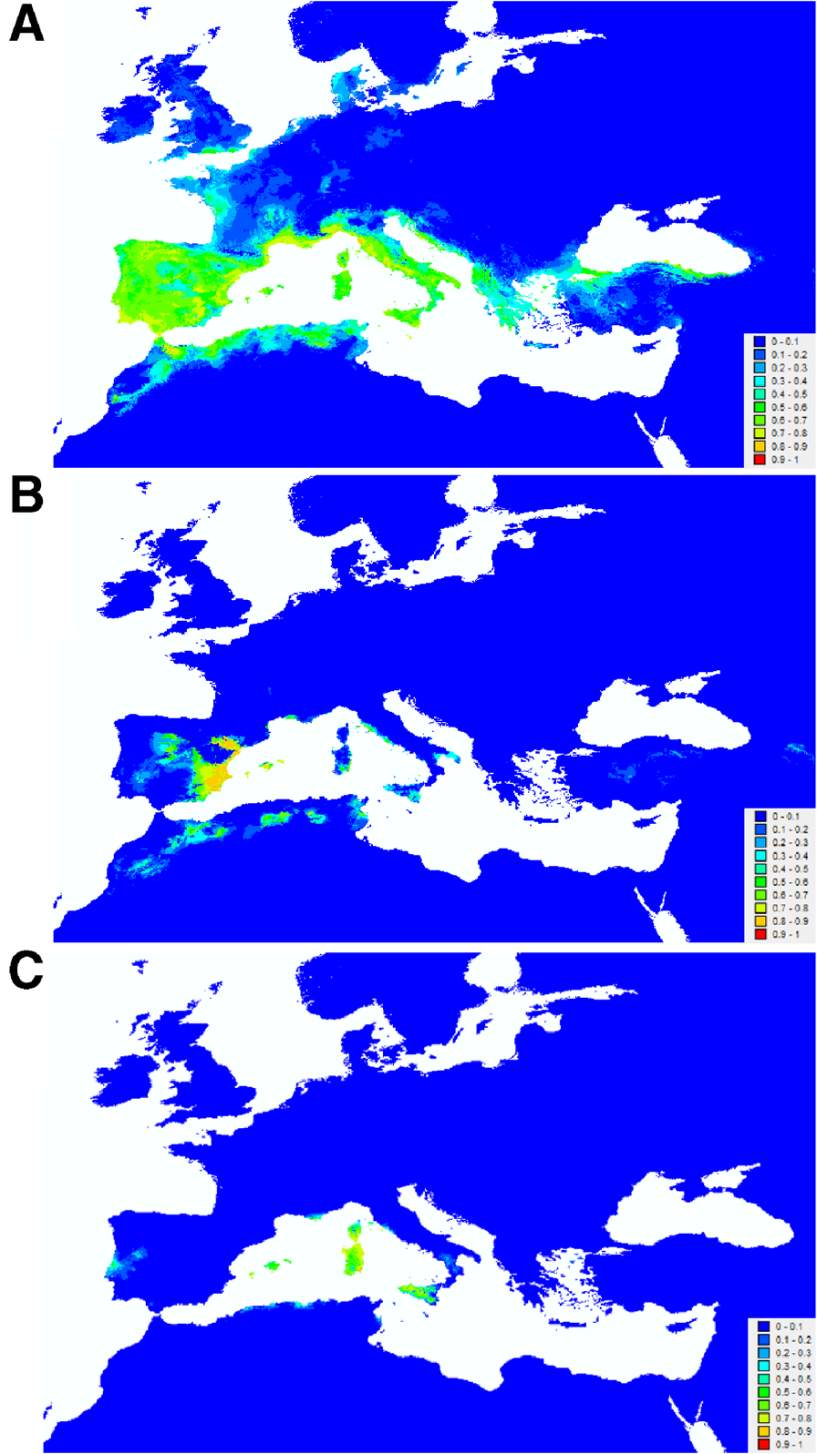
**Environmental niche models (ENMs) using MaxEnt for A)** ***A. brunneus*** **; B)** ***A. ramblae*** **; and C)** ***A. rufulus*** **.** Higher MaxEnt probabilities (red colours) represent areas more suitable for the species according to the MaxEnt models, lower values (blue) represent less suitable areas. These continuous measures of habitat suitability were used in ENM-based tests of niche similarity.

**Figure 7:**
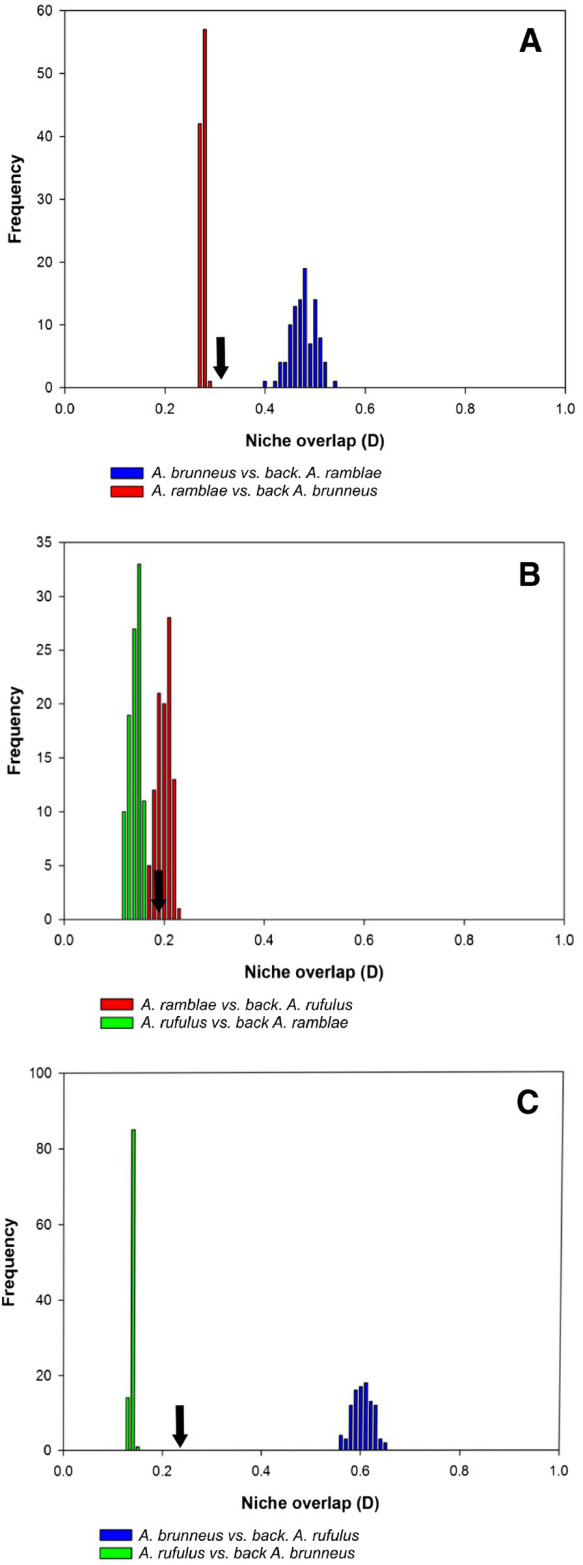
**Background similarity test results for species within the** ***Agabus brunneus*** **species complex.** Observed niche overlap values (arrows) were compared with null distributions (100 replicates) generated by comparing model suitability values of one species to those generated from random cells drawn from the background area of the other species.

Results of ENM of each species, estimated with MaxEnt, had low spatial overlap. The degree of niche overlap estimated by Schoener’s D statistic was lower than 0.373 for all pairwise comparisons between the three species, suggesting differences in the climatic niche among them ([52]; Figure 6 and Figure 7). The null hypothesis of no differences in ecological niches explained by environmental differences between areas of occupancy alone was accepted (lower p-value = 0.17) for the comparison between *A. ramblae* and *A. rufulus* (despite a low D value, 0.20, this was not significantly more different than expected by chance, Figure 7), and rejected (p < 0.05) for comparisons between *A. brunneus* and the other two species.

**Figure 8:**
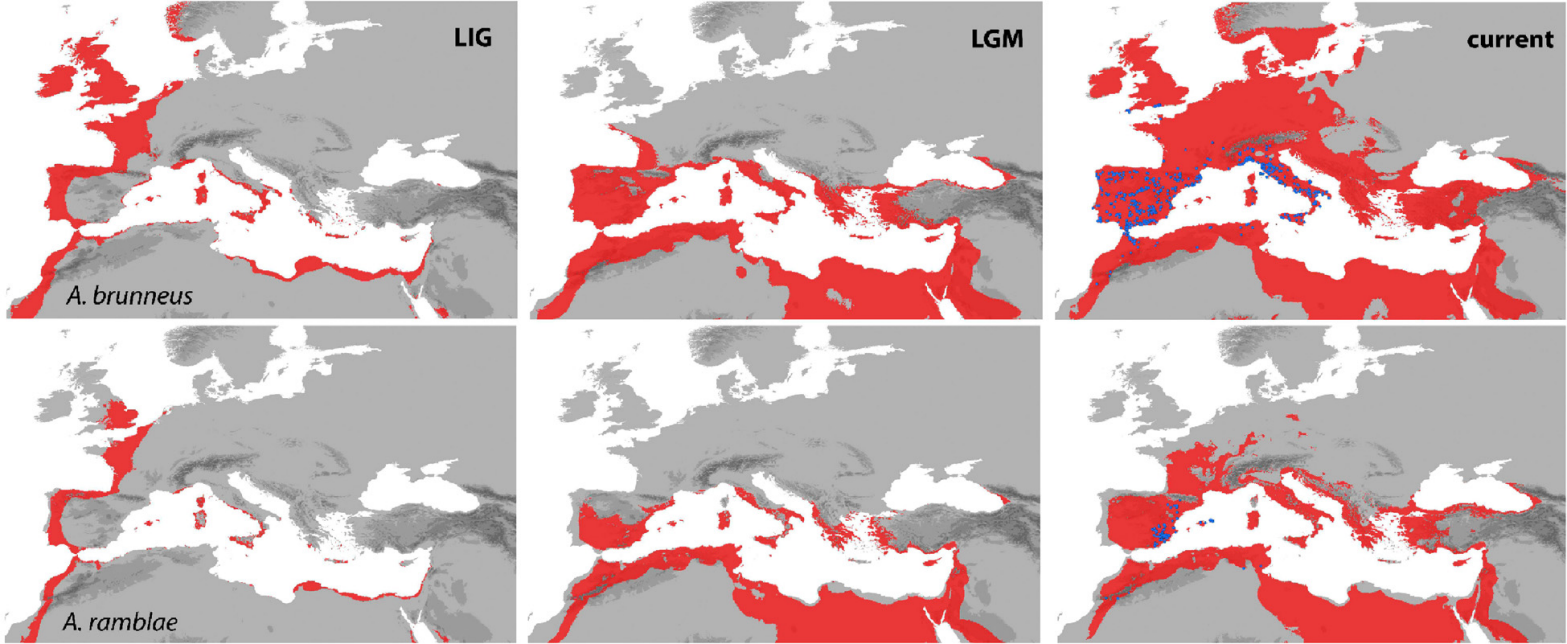
**Estimated potential distribution for** ***A. brunneus*** **and** ***A. ramblae*** **during the last interglacial (LIG), last Glacial Maximum (LGM) and the present.** In red, areas considered to be climatically suitable for the species (according to the ecological conditions of the localities in which they are currently found). Blue dots, current localities of the species.

The potential distributions of *A. brunneus* and *A. ramblae* during the last interglacial (LIG), reconstructed using a climatic envelope, were similar but far more restricted than their current distributions, with most of the Iberian peninsula and North Africa considered unsuitable (Figure 8). This was especially the case for *A. ramblae*, for which, in terms of current range, only the Balearic Islands and some areas in the High Atlas in Morocco appeared appropriate (Figure 8). The largest difference between the LIG reconstruction and the current climate for the studied variables was seasonality, with the areas considered to be unsuitable having values beyond those of their current ranges (Additional file 15: Figure S8).

During the last glacial maximum (LGM) the potential distribution of the two species increased to cover most of their current ranges: for *A. ramblae* only the northernmost known locality was outside the potential LGM distribution (Alcampell, in the province of Huesca), which was also an outlier in the representation of the climatic niche of the species (Additional file 14: Figure S7). For *A. brunneus* some Pyrenean localities, those on the north coast of France and the south coast of Britain, north Italy and the French Massif Central were outside the LGM potential distribution (Figure 8), but most of its current distribution corresponded to its potential range in the LGM. On the contrary, for both species the estimated potential present day distribution was much wider than the actual range: for *A. brunneus* it included the whole Mediterranean area and most of Europe, and for *A. ramblae* most of the Mediterranean and central France (Figure 8).

## Discussion

### Species limits within the *A. brunneus* complex – morphology, molecules and physiology

Our initial criterion for species recognition was the morphology of the aedeagus, in agreement with current taxonomy [16]. The simple measures used were able to unambiguously discriminate between *A. brunneus* and *A. ramblae*, but *A. rufulus*, the insular species, had an intermediate morphology for these characters, although the shape of the aedeagus in lateral view allows the unequivocal identification of this species [16]. The three species were clearly recovered as monophyletic with the nuclear marker (*H3*), with the exception of one female from the southeast of the Iberian Peninsula, characterised as *A. ramblae* but nested with other peninsular *A. brunneus*, which may represent a misclassified individual (in the same area both *A. brunneus* and *A. ramblae* can be found, Additional file 4: Table S2). In contrast to nuclear genes, the mitochondrial markers recovered a paraphyletic *A. ramblae* as ancestral to *A. brunneus* and *A. rufulus.* This paraphyly could be due to incomplete lineage sorting resulting from the recent evolution of the group [62–64], which is estimated to have diverged mostly within the last 0.5 MY –an insufficient time to reach reciprocal monophyly. However, in our uncalibrated tree for Agabini the estimated rate of *cox1* was approximately eight times higher than that of *H3*, which being nuclear, should have a longer coalescent time than mitochondrial genes [65]. A possible explanation could be a complete replacement of the *A. ramblae* mitochondrial genome by that of *A. brunneus* in the Iberian peninsula, due to an early introgression event which did not result in phenotypic change between North African and Iberian populations of *A. ramblae* (either morphological, ecological or in thermal tolerance). The clear morphological separation between the two species argues against continued events of gene flow between them, which if present should have produced a higher frequency of specimens with intermediate morphologies. But given the low variability among the species of the *A. brunneus* complex in the *H3* fragment, this difference in coalescent times could also be simply due to random effects.

There is some additional evidence of a mismatch between morphology and some of the genetic markers used that suggests occasional introgression, but this is only seen in geographically marginal areas: Sardinia (between *A. rufulus* and *A. brunneus*), Menorca (between *A. ramblae* and *A. brunneus*), and possibly Tunisia (also between *A ramblae* and *A. brunneus*). Mitochondrial haplotypes of *A. brunneus* were found in individuals identified by morphology and nDNA as *A. ramblae* in Menorca, and *A. rufulus* in Sardinia. This could be due to a secondary colonisation of the islands by continental *A. brunneus*, similarly to what has been described in a related genus of diving beetle (*Meladema*) in the Canary Islands [66]. In Tunisia, southern populations were characterized as *A. ramblae* by both morphology and nDNA, but the mtDNA clustered with *A. brunneus* and Iberian haplotypes of *A. ramblae*. Two possible scenarios may account for such results: Tunisian *A. ramblae* could have arrived directly from the Iberian peninsula, which seems unlikely given the geographical distance and the presence of sea barriers; or they could have arrived from elsewhere in North Africa, and then hybridised with northern *A. brunneus* either also from North Africa or from Sicily. A Moroccan origin is supported by the presence of other water beetles typical of arid or saline habitats with a similar distribution through central and south Morocco to south Tunisia, such as *Enochrus risii* Arribas et al. [67] or *Ochthebius salinator* Peyerimhoff [68]. The situation of *A. ramblae* in the Iberian peninsula is more complex and difficult to interpret: some specimens had mitochondrial haplotypes clustering with those of *A. ramblae* from Morocco, suggesting the persistence within Iberia of some of the ancestral Moroccan haplotypes and a derived origin of most of both the Iberian *A. ramblae* and *A. brunneus*. But again, the replacement of the *A. ramblae* mitochondrial genome by that of *A. brunneus* through introgression in secondary contact zones cannot be discarded.

The two species with broadly overlapping ranges, *A. brunneus* and *A. ramblae*, were also ecologically different as measured through ecological niche modelling and the background test. Our experimental results show that the greater resistance of *A. brunneus* to lower temperatures may have been a key feature to allow its range expansion during the LGM (see below). This difference was reflected in the significantly lower minimum temperature of the coldest month of the places in which *A. brunneus* is currently found. This species had also significant differences in thermotolerance between populations, with the northern one (with an average lowest temperature of the coldest month of 2.8 °C) being more resistant to cold than the southern population (average lowest temperature of the coldest month of 6.0 °C). With our data it is not possible to discriminate whether this difference results from local adaptation or phenotypic plasticity, although Calosi et al. [17] found that members of the group did not significantly adjust their LTL after a short period of acclimation in the laboratory. This suggests that *A. brunneus* populations may instead adapt to local temperature conditions, these evolutionary changes possibly facilitating range expansions.

### Evolutionary history of the *A. brunneus* complex

We found strong support for the monophyly of the *A. brunneus* complex, and also recovered a monophyletic *A. brunneus* group, albeit with lower support. The long stem branch of the complex is atypical within the rest of Agabini, and as there are no known species worldwide that could be more closely related to it [28], it appears that the *A. brunneus* complex has a very isolated position amongst extant Agabini.

Although the support for the internal relationships of the *A. brunneus* complex was low, the selected mitochondrial topology recovered the Moroccan populations of *A. ramblae* as paraphyletic, suggesting an origin of the complex in western North Africa, with colonization of the Iberian peninsula ca. 0.5 Ma. As seen above, much of the likely recent introgression between the species of the group is restricted to a few populations in secondary contact zones, so we do not expect this to affect the mitochondrial phylogeny. Even if some paraphyly was due to early introgression within the Iberian peninsula this would not affect our biogeographic scenario. The colonization of the Balearic Islands and Corsica and Sardinia happened in a narrow temporal window, possibly from Iberia. Although we cannot discard a direct colonization from North Africa, this seems less plausible due to the longer geographical distances involved. According to our estimation, *A. brunneus* split from SE Iberian *A. ramblae* ca. 0.25 Ma. An alternative scenario is that *A. brunneus* originated in Morocco and colonized the Iberian Peninsula in parallel with *A. ramblae*, but as well as being less parsimonious this hypothesis seems less plausible since some Iberian *A. ramblae* have mitochondrial haplotypes clustering with those from Morocco.

The ecological and physiological differences between *A. brunneus* and *A. ramblae* may have originated during the speciation process or evolved later, with an initial separation only due to isolation. Either way, at some point *A. brunneus* acquired the capacity to resist colder temperatures.

The demographic analyses estimated a population expansion of the complex at the start of the last glaciation 30–40,000 YBP, in agreement with the extension of their potential distributions during the last glacial maximum (LGM, 21,000 YBP). For both widespread species, potential distributions during the LGM covered practically the totality of their current ranges, and were mostly determined by minimum temperatures and climatic seasonality. It is remarkable that only very few current known localities (mostly for *A. brunneus*) are outside the reconstructed potential range of both species during the LGM, despite a large increase in apparently suitable geographical areas both in Europe and north Africa. During the LGM sea levels could have been up to 200 m lower, probably extending the suitable surface and potentially favouring the expansion of the continental species of the group to areas now isolated by sea barriers, such as south Britain.

The current absence of *A. brunneus* from central and northern Europe cannot be attributed to the effect of anthropogenic habitat modification, as there are no historic or Quaternary fossil records of the species outside its current range [24]. The external appearance of species of the *A. brunneus* complex is very characteristic, so that even incomplete remains would be recognizable, and in central and northern Europe the fossil record from the LGM and the Holocene is very complete [24]. Possible explanations for the absence of range expansion in *A. brunneus* and *A. ramblae* after the LGM are the presence of undetected climatic, biotic or ecological limiting factors, or simply a lack of sufficient time for these species to arrive at equilibrium with their potential ranges. All species of the *A. brunneus* complex are exclusively found in running waters, and such lotic taxa have, in general, weaker dispersal abilities than their lentic relatives, leading to a stronger mismatch between their realized and potential distributions in central and north Europe [66,69].

## Conclusions

Using a combination of morphology, genetics, ecological niche modelling of current and paleoecological data and physiological experiments we have reconstructed the surprisingly complex evolutionary history of this diving beetle clade in the western Mediterranean. The *A. brunneus* complex diversified ca. 0.6-0.25 Ma, most likely in the south of the Iberian peninsula after the colonization of *A. ramblae* from north Morocco. Whilst insular populations (*A. ramblae* in the Balearic Islands and *A. rufulus* in Corsica and Sardinia) did not apparently differentiate substantially in either morphology or ecology, continental *A. brunneus* evolved the most distinctive morphology within the complex, as well as wider tolerance to cold habitats, something that seems to have facilitated range expansion.

From a reduced potential distribution during the LIG, *A. brunneus* and *A. ramblae* appear to have expanded their ranges during the last glacial (0.03-0.01 Ma) (*A. brunneus* to a much wider area), covering most of their LGM potential rages in the western Mediterranean. This expansion was accompanied by a population expansion, as identified through demographic models. However, despite much wider current potential distributions, both species have not occupied areas beyond their LGM potential distribution except for some isolated populations of *A. brunneus* in France and England. In Sardinia, the Balearic Islands and possibly Tunisia, secondary contact between species of the complex has resulted in introgression, with some specimens showing discordance between mitochondrial haplotypes typical of *A. brunneus* and nuclear sequences and morphology typical of *A. rufulus* or *A. ramblae* respectively.

Our work highlights the complex dynamics of speciation and range expansions within refugia during the last glacial cycle, and the fact that the biota of southern Europe, in addition to being a source of colonisers of formerly glaciated areas in the north, experienced much evolutionary change during this time period. It also highlights the fundamental but often neglected role of North Africa as source of biodiversity in Europe [70–74].

## Availability of supporting data

All raw data are included in the Supplementary files with the exception of the sequences, deposited in the EMBL database with accession numbers LM654767-LM655064 and LM655068-LM655168.

## Additional files

Additional file 1: Figure S1. Measures used for the identification of the specimens. A) Median lobe of the aedeagus of *A. ramblae* in ventral view, with the measurements used. The global measure of asymmetry used was AD = RD-LD. B) Maximum body length (BL, excluding head) and width (BW). Additional file 2: Table S1. List of specimens with morphometric measurements. Ref., reference of the specimen: with AH and AI, voucher numbers of extracted specimens (see Additional file 4: Table S2); BM, other material not used for DNA extraction. BL, body length (from anterior side of prototum to apex of elytra); BW, maximum body width; AL, length of the aedeagus; AD, asymmetry of the aedeagus (see Additional file 1: Figure S1). Additional file 3: Table S5. Thermal tolerance data. Population: SPA-Cádiz: Spain, Cádiz (data from ref.[17]); SPA-Girona: Spain, Girona (42°08'48''N 2°34'48''E); SPA-Murcia: Spain, Murcia (data from ref.[17]); MOR-Tinghir: Morocco, Tinghir (31°33'25''N 5°34'49''W); ITA-Sardinia: Italy, Sardinia (data from ref.[17]). Additional file 4: Table S2. List of the specimens used for DNA extraction, with accession numbers of the sequences. Extraction method: Charge, Charge Switch gDNA Tissue Kit (Invitrogen, Carlsbad, USA); Invisorb, Invisorb Spin DNA Extraction Kit (Invitek GmbH, Berlin, Germany); Qiagen, Qiagen DNeasy Tissue Kit (Qiagen GmbH, Hilden Germany). In the column 16S, with asterisk: single fragment including the 3’ end of *rrnL*, the full *trnL* and the 5’ end of *nad1*; in the rest either the fragment is divided in two sequences or includes only the *rrnL* fragment. Additional file 5: Table S3. List of primers used and PCR conditions. Additional file 6: Table S4. Climatic variables used in the niche modelling. Additional file 7: Table S6. Localities used for the niche modelling of the three species. BD (BIODIV), unpublished database of the Department of Ecology and Hydrology, University of Murcia (Spain); CKmap, Checklist and distribution of Italian fauna (http://www.faunaitalia.it/ckmap/); ESACIB, database “Escarabajos Acuáticos Ibéricos” (Sánchez-Fernández et al. 2008); GBIF, Global Biodiversity Information Facility (http://www.gbif.org); NBN Gateway, National Biodiversity Network of United Kingdom (http://www.nbn.org.uk). Additional file 8: Figure S2. Background area used for each species in the background similarity test. A) *Agabus brunneus*; B) *A. ramblae*; C) *A. rufulus*. Additional file 9 Text S1. Method used to obtain the potential distribution of species. Additional file 10: Figure S3. Bivariant plot of the measures of the median lobe of the aedeagus. Open circles, *Agabus ramblae*; grey diamonds, *A. brunneus*; black triangles, *A. rufulus*. Open square: male *A. rufulus* from Sardinia with an *A. brunneus* mitochondrial haplotype. Additional file 11: Figure S4. Ultrametric tree of Agabini obtained in BEAST using only mDNA data, constraining the monophyly of the ingroup and outgroup (genus *Platynectes*), the genera and the *A. brunneus* complex. To calibrate the tree we used an a-priori rate of 0.01 substitutions/site/MY (see text for details). Numbers in nodes in black font, posterior probabilities (above 0.5); in red, estimated age. Additional file 12: Figure S5. Phylogenetic analyses of the *cox1* data. Calibrated tree obtained in BEAST, using a mean rate of 0.02+/−0.001 substitutions/site/MY. Small numbers in nodes, estimated age (Ma), large numbers in nodes, posterior Bayesian probabilities (pp). Negative branches collapsed in polytomies. In red, specimens likely to have introgressed mitochondrial DNA from *A. brunneus*. Additional file 13: Figure S6. Phylogram obtained in RAxML with the H3 sequences. Numbers in nodes, posterior probabilities obtained in BEAST (if above 0.5) / bootstrap support (if above 50%). With an asterisk, female from Albacete (SE Spain) of uncertain identity. Additional file 14: Figure S7. Representation of the scores of the two first axis of the PCA with all climatic variables. Grey surface, climatic space of the western Palaeartic (background). In colours, climatic space occupied by the three species. Additional file 15: Figure S8. Reconstructed seasonality and minimum temperature of the coldest month during the last glacial interval (upper row) and the last glacial maximum (lower row). Blue circles, current distribution of *A. ramblae*.
