# Supplementary figures and images for: Thermal niche evolution and geographical range expansion in a species complex of western Mediterranean diving beetles
Source: BMC Evol Biol. 2014 Sep 4;14:187. doi: 10.1186/s12862-014-0187-y (PMC4180321; doi:10.1186/s12862-014-0187-y)

A

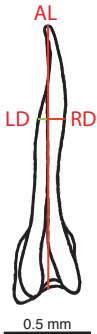

B

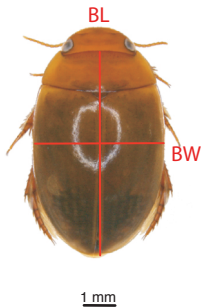

Supplement: Additional file 1: Figure S1. — Measures used for the identification of the specimens. A) Median lobe of the aedeagus of A. ramblae in ventral view, with the measurements used. The global measure of asymmetry used was AD = RD-LD. B) Maximum body length (BL, excluding head) and width (BW). [file 12862_2014_187_MOESM1_ESM.pdf]

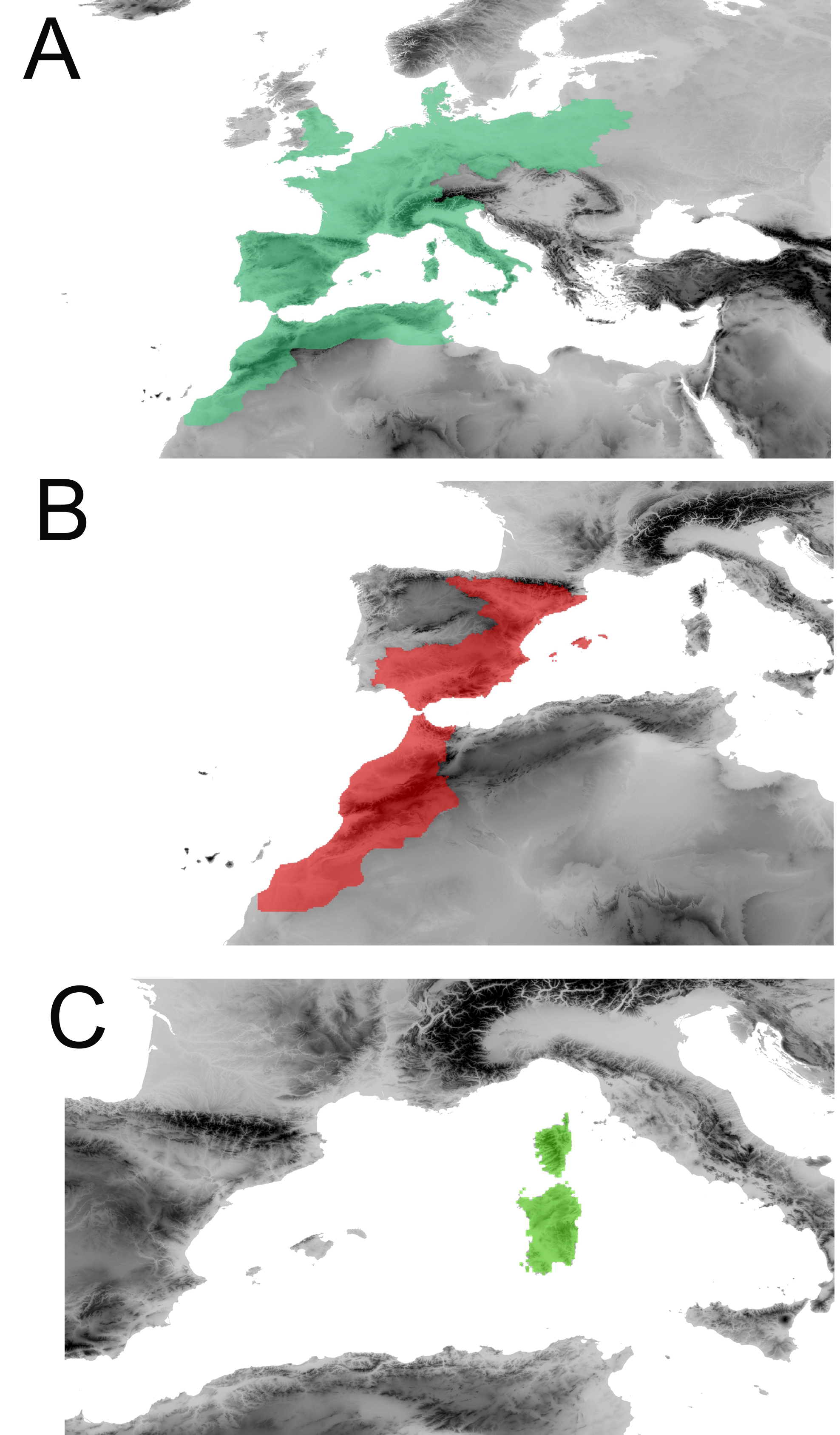

Supplement: Additional file 8: Figure S2. — Background area used for each species in the background similarity test. A) Agabus brunneus; B) A. ramblae; C) A. rufulus. [file 12862_2014_187_MOESM8_ESM.jpeg]

Aedeagus maximum deviation (AD) (mm)

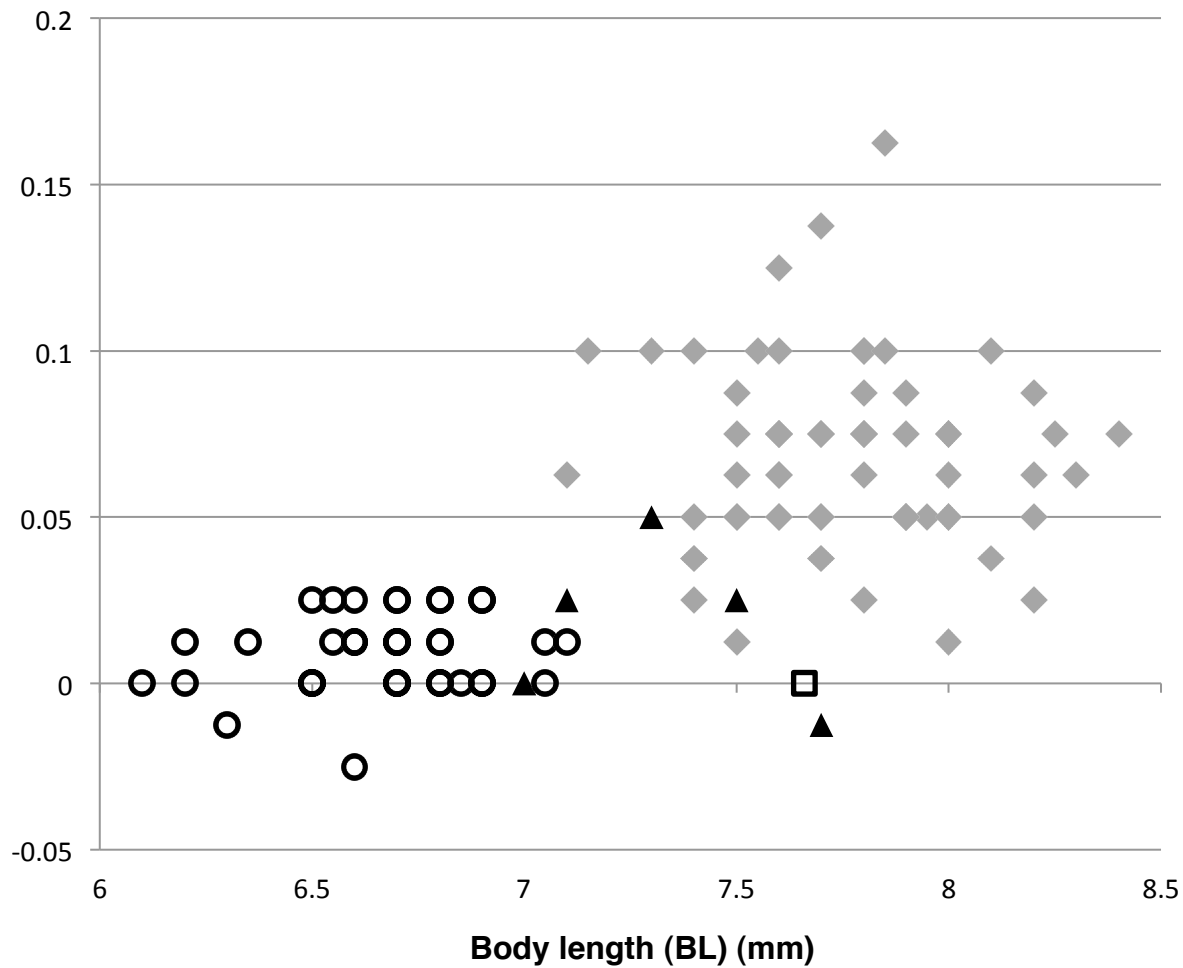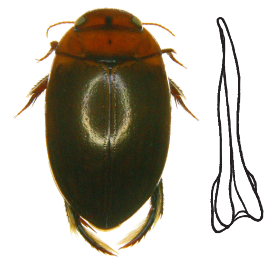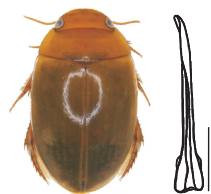

Supplement: Additional file 10: Figure S3. — Bivariant plot of the measures of the median lobe of the aedeagus. Open circles, Agabus ramblae; grey diamonds, A. brunneus; black triangles, A. rufulus. Open square: male A. rufulus from Sardinia with an A. brunneus mitochondrial haplotype. [file 12862_2014_187_MOESM10_ESM.pdf]

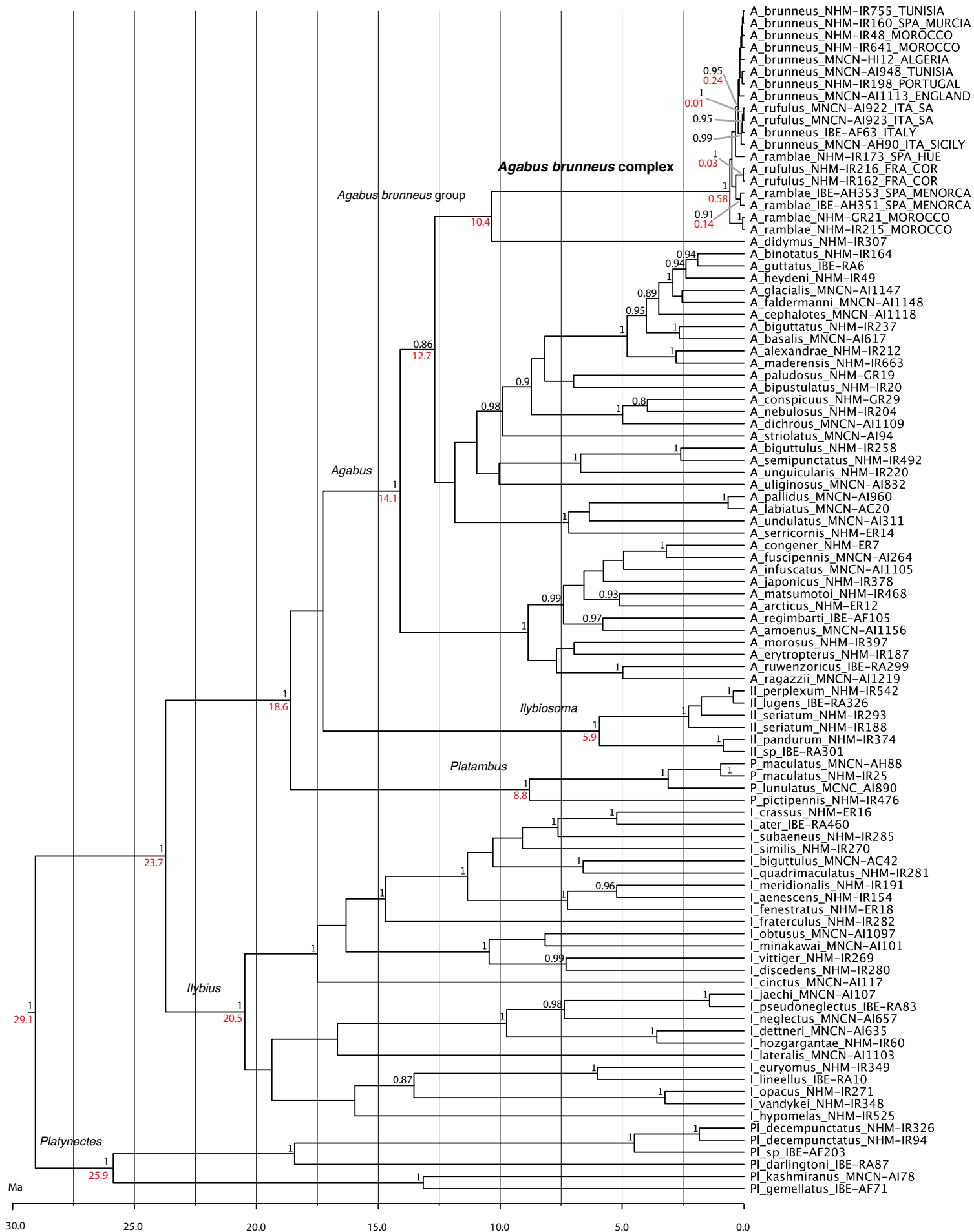

Supplement: Additional file 11: Figure S4. — Ultrametric tree of Agabini obtained in BEAST using only mDNA data, constraining the monophyly of the ingroup and outgroup (genus Platynectes), the genera and the A. brunneus complex. To calibrate the tree we used an a-priori rate of 0.01 substitutions/site/MY (see text for details). Numbers in nodes in black font, posterior probabilities (above 0.5); in red, estimated age. [file 12862_2014_187_MOESM11_ESM.pdf]

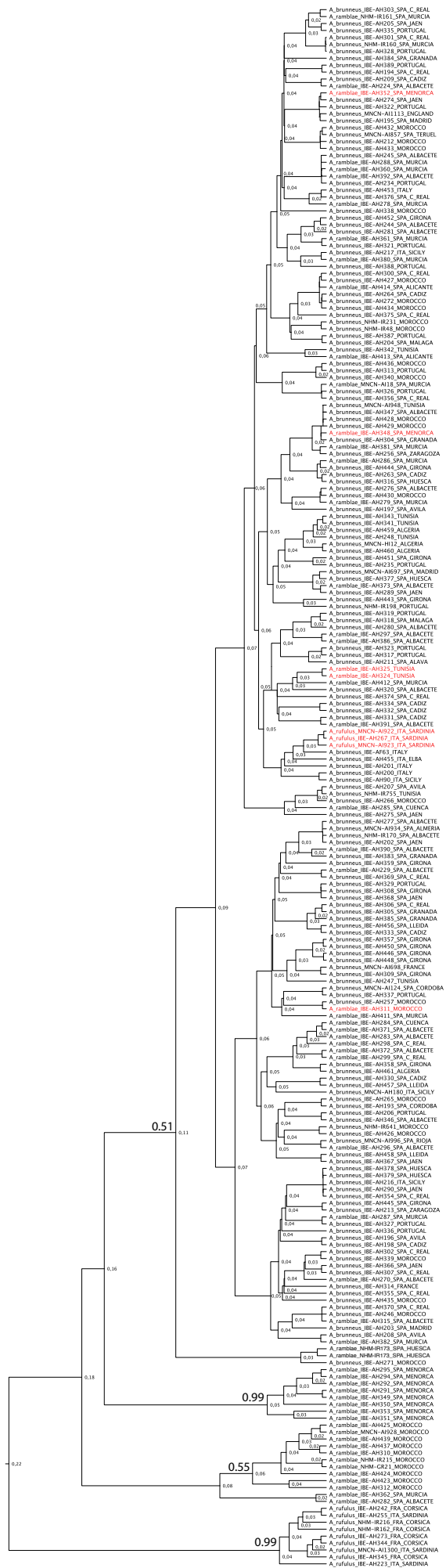

Supplement: Additional file 12: Figure S5. — Phylogenetic analyses of the cox1 data. Calibrated tree obtained in BEAST, using a mean rate of 0.02+/−0.001 substitutions/site/MY. Small numbers in nodes, estimated age (Ma), large numbers in nodes, posterior Bayesian probabilities (pp). Negative branches collapsed in polytomies. In red, specimens likely to have introgressed mitochondrial DNA from A. brunneus. [file 12862_2014_187_MOESM12_ESM.pdf]

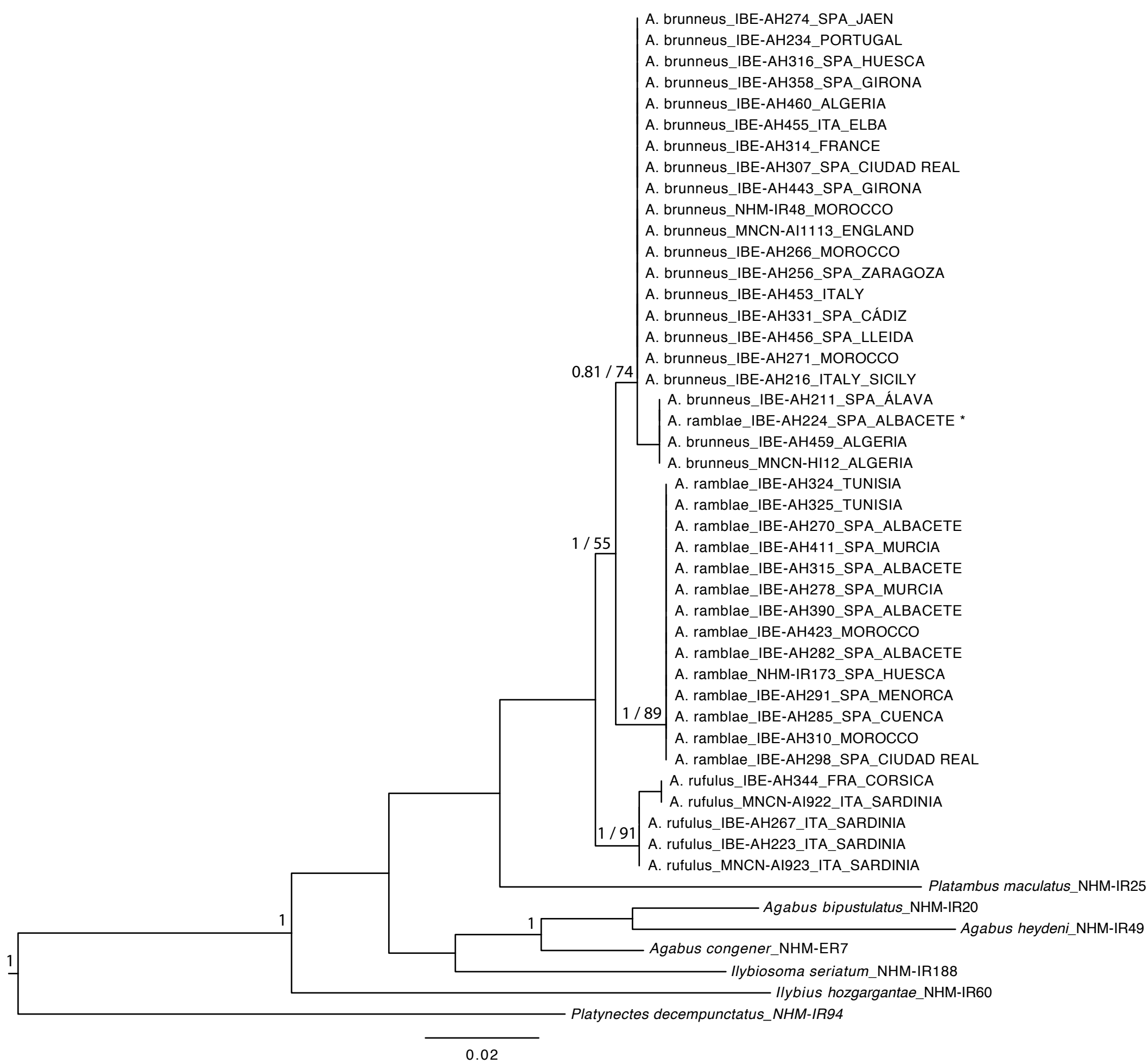

Supplement: Additional file 13: Figure S6. — Phylogram obtained in RAxML with the H3 sequences. Numbers in nodes, posterior probabilities obtained in BEAST (if above 0.5) / bootstrap support (if above 50%). With an asterisk, female from Albacete (SE Spain) of uncertain identity. [file 12862_2014_187_MOESM13_ESM.pdf]

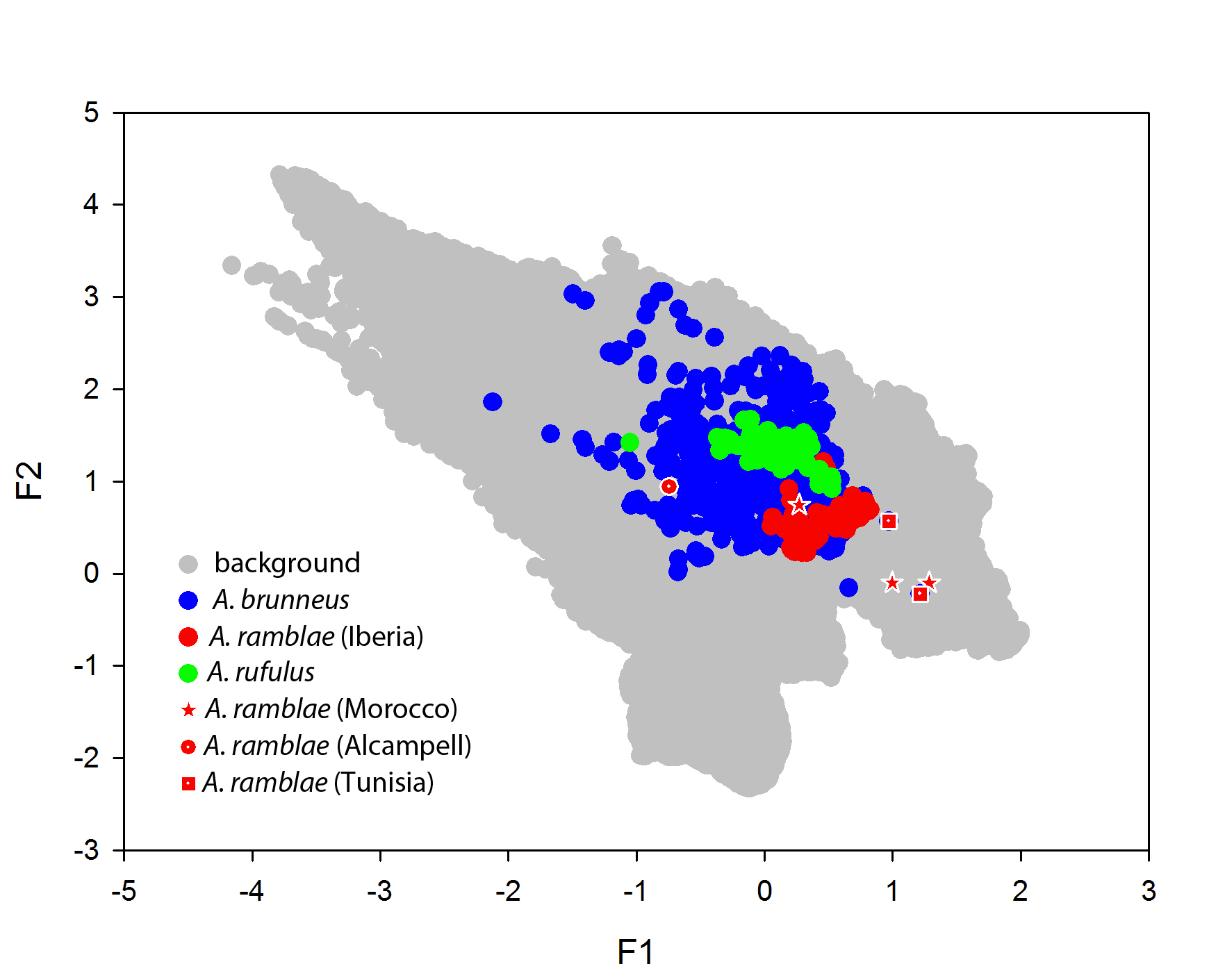

Supplement: Additional file 14: Figure S7. — Representation of the scores of the two first axis of the PCA with all climatic variables. Grey surface, climatic space of the western Palaeartic (background). In colours, climatic space occupied by the three species. [file 12862_2014_187_MOESM14_ESM.jpeg]

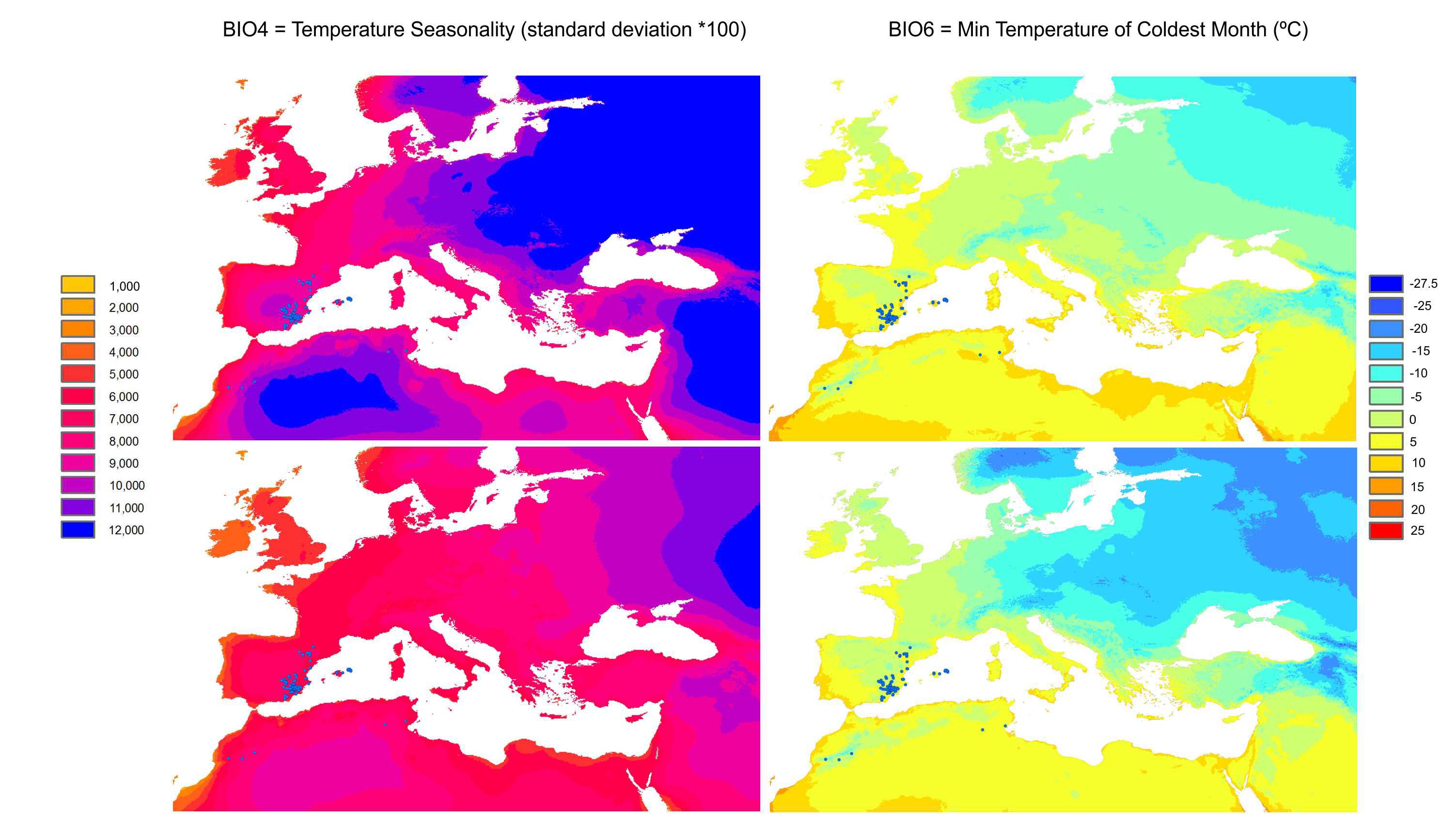

Supplement: Additional file 15: Figure S8. — Reconstructed seasonality and minimum temperature of the coldest month during the last glacial interval (upper row) and the last glacial maximum (lower row). Blue circles, current distribution of A. ramblae. [file 12862_2014_187_MOESM15_ESM.jpeg]
