# Supplementary material for: Thermal niche evolution and geographical range expansion in a species complex of western Mediterranean diving beetles
Source: BMC Evol Biol. 2014 Sep 4;14:187. doi: 10.1186/s12862-014-0187-y (PMC4180321; doi:10.1186/s12862-014-0187-y)
Supplement: Additional file 9 Text S1. — Method used to obtain the potential distribution of species. [file 12862_2014_187_MOESM9_ESM.docx]

**Thermal niche evolution and geographical range expansion in a species complex of western Mediterranean diving beetles.**

**Amparo Hidalgo-Galiana, David Sánchez-Fernández, David T. Bilton, Alexandra Cieslak and Ignacio Ribera**

**Additional file 9: Text S1. Method used to obtain the potential distribution of species.**

 Assuming that presence localities reflect a subset of the suitable conditions under which a species can maintain viable populations, environmental envelope is an approach directed at maximizing the capacity to represent geographically the potential distribution of species when they are only based on distributional data (see Jiménez-Valverde et al., 2011; Sánchez-Fernández et al., 2013 for an application of this procedure). In this procedure, the maximum and minimum scores (extreme values) for all relevant climatic variables from the entire set of observed presence cells are first calculated for each species. Then, all grid cells with climatic values falling within the mentioned range are designated as suitable, and all cells outside it as unsuitable. In this way, the extreme values are used to derive a binary distributional hypothesis about the areas having climatically suitable conditions (climatic potential distribution), under the assumption that recorded occurrences reflect the spectrum of climatic conditions in which the species can We consider relevant variables as the minimum set of climatic variables needed to explain the occurrence of each species as estimated using an ecological-niche factor analysis (ENFA; Hirzel et al., 2002; Basille et al., 2008). Factors were retained or discarded based on their eigenvalues relative to a broken-stick distribution (Hirzel et al., 2002). Climatic variables selected as relevant were those showing the highest correlation values with the retained ENFA factors. In our case, the same four variables (Temperature Seasonality; Max. Temp of the warmest month; Min. Temp of the coldest month and Thermal Annual Range) were selected for both species.

**References**

Basille, M., Calenge, C., Marboutin, E., Andersen, R. & Gaillard, J.M. (2008) Assessing habitat selection using multivariate statistics: some refinements of the ecological-niche factor analysis. *Ecological Modelling*, **211**, 233–240.

Hirzel, A.H., Hausser, J., Chessel, D. & Perrin, N. (2002) Ecological-niche factor analysis: how to compute habitat suitability maps without absence data? *Ecology*, **83**, 2027–2036.

Jiménez-Valverde, A., Peterson, A.T., Soberón, J., Overton, J., Aragón, P. & Lobo, J.M. (2011) Use of niche models in invasive species risk assessments. *Biological Invasions*, **13**, 2785–2797.

Sánchez-Fernández, D.; Abellán, P.; Picazo, F.; Millán, A.; Ribera, I. & Lobo, J.M. (2013) Do protected areas represent species’ optimal climatic conditions? *Diversity and Distributions*, **19**, 1407-1417.
